# [Retracted] Exogenous hydrogen sulfide protects against high glucose‑induced apoptosis and oxidative stress by inhibiting the STAT3/HIF‑1α pathway in H9c2 cardiomyocytes

**DOI:** 10.3892/etm.2024.12479

**Published:** 2024-03-11

**Authors:** Jing Li, Yi-Qiang Yuan, Li Zhang, Hua Zhang, Shen-Wei Zhang, Yu Zhang, Xue-Xi Xuan, Ming-Jie Wang, Jin-Ying Zhang

Exp Ther Med 18:3948–3958, 2019; DOI: 10.3892/etm.2019.8036

Following the publication of this paper, it was drawn to the Editors’ attention by a concerned reader that certain of the cell apoptotic data shown in Fig. 4A on p. 3953 were strikingly similar to data appearing in different form in other articles written by different authors at different research institutes that had already been published elsewhere prior to the submission of this paper to *Experimental and Therapeutic Medicine* (one of which has been retracted). In view of the fact that the above mentioned data had already apparently been published previously, the Editor of *Experimental and Therapeutic Medicine* has decided that this paper should be retracted from the Journal. The authors were asked for an explanation to account for these concerns, but the Editorial Office did not receive a reply. The Editor apologizes to the readership for any inconvenience caused.

